# Efficacy and safety of continuous veno-venous hemofiltration for acute respiratory distress syndrome: a meta-analysis

**DOI:** 10.3389/fmed.2026.1856590

**Published:** 2026-07-17

**Authors:** Rui Wang, Sicheng Xu

**Affiliations:** 1 Xinjiang Medical University, Urumqi, China; 2 The First Affiliated Hospital of Xinjiang Medical University, Urumqi, China

**Keywords:** acute respiratory distress syndrome, critical care, efficacy, meta-analysis, veno-venous hemofiltration

## Abstract

**Background:**

To evaluate the efficacy and safety of continuous veno-venous hemofiltration (CVVH) in patients with acute respiratory distress syndrome (ARDS) using a meta-analytic approach.

**Methods:**

Randomized controlled trials on CVVH for ARDS were retrieved from Chinese databases (Wanfang, CNKI) as well as PubMed and Web of Science. Meta-analysis was performed using Review Manager 5.3 software on the extracted data.

**Results:**

This meta-analysis included a total of 28 randomized controlled trials (RCTs) involving 1,739 patients with ARDS, comprising 897 cases in the CVVH group and 842 cases in the conventional treatment group. The results demonstrated that the CVVH group exhibited superior outcomes compared to the conventional treatment group in terms of oxygenation index (MD = 52.80, 95% CI [39.75, 65.84], *p* < 0.00001), mortality rate (OR = 0.43, 95% CI [0.34, 0.56], *p* < 0.00001), ICU hospitalization duration (MD = −4.88, 95% CI [−6.45, −3.31], *p* < 0.00001), mechanical ventilation duration (MD = −4.10, 95% CI [−5.04, −3.15], *p* < 0.00001), APACHE II score (MD = −3.62, 95% CI [−4.30, −2.95], *p* < 0.00001), VAP incidence (OR = 0.21, 95% CI [0.12, 0.36], *p* < 0.00001), PaO₂ (MD = 7.49, 95% CI [5.31, 9.67], *p* < 0.00001), PEEP (MD = −2.07, 95% CI [−2.85, −1.29], *p* < 0.00001), TNF-*α* (MD = −19.17, 95% CI [−27.77, −10.56], *p* < 0.0001), IL-6 (MD = −24.86, 95% CI [−31.88, −17.84], *p* < 0.00001), CRP (MD = −23.48, 95% CI [−39.96, −7.00], *p* = 0.005), PCT (MD = −2.31, 95% CI [−3.78, −0.84], *p* = 0.002), WBC (MD = −3.36, 95% CI [−6.30, −0.42], *p* = 0.03), MAP (MD = 3.57, 95% CI [0.55, 6.58], *p* = 0.02), HR (MD = −11.72, 95% CI [−14.07, −9.38], *p* < 0.00001), and EVLWI (MD = −2.61, 95% CI [−4.60, −0.62], *p* < 0.00001). No statistically significant differences were observed between the two groups regarding PaCO₂ (MD = −3.05, 95% CI [−7.12, 1.02], *p* = 0.14) or cardiac index (CI) (MD = 0.36, 95% CI [−0.89, 1.60], *p* = 0.57).

**Conclusion:**

In conclusion, CVVH as an adjunctive therapy for ARDS improves oxygenation, reduces mortality, shortens ICU and ventilator days, lowers APACHE II score and heart rate, decreases VAP incidence, and exerts anti-inflammatory and fluid-removing effects. Although substantial heterogeneity and potential publication bias exist (e.g., for oxygenation index and mechanical ventilation duration), and all included studies are from China, CVVH demonstrates promising clinical value. Large-scale, multicenter RCTs are needed to confirm these findings and optimize treatment protocols.

## 1 Introduction

Acute respiratory distress syndrome (ARDS) is a condition characterized by increased permeability of the alveolar-capillary barrier formed by microvascular endothelium and alveolar epithelium, leading to extravascular accumulation of protein-rich edema fluid, leukocytes, and red blood cells within the alveolar space, along with production of pro-inflammatory cytokines such as tumor necrosis factor-*α*. Clinically, it manifests as refractory hypoxemia and non-cardiogenic pulmonary edema (1, 2). Both the incidence and mortality rates of this disease are high, with approximately 3 million patients diagnosed with acute respiratory distress syndrome (ARDS) globally each year (3). The hospitalization mortality rates for mild, moderate, and severe acute respiratory distress syndrome were 34.9, 40.3, and 46.1%, respectively (4, 5). Furthermore, only approximately one-third of patients meeting the diagnostic criteria can be identified by clinicians.

Current treatments for ARDS primarily include mechanical ventilation, restriction of fluid intake, and corticosteroids, although each approach has certain limitations (5). In recent years, numerous domestic and international studies have reported that continuous renal replacement therapy (CRRT), initially primarily used for treating patients with renal failure, has gradually become an essential adjunctive treatment for acute respiratory distress syndrome (ARDS) (4). However, all currently employed CRRT modalities utilize an extracorporeal veno-venous circuit. Depending on the solute clearance mechanism, these therapies include continuous veno-venous hemofiltration (CVVH), which primarily relies on convection for solute removal; continuous veno-venous hemodialysis (CVVHD), which depends mainly on diffusion; and continuous veno-venous hemodiafiltration (CVVHDF), which combines both convection and diffusion mechanisms (6). Compared to diffusion, convection (blood filtration) may be more effective in removing medium molecular weight solutes such as cytokines (7). The primary clinical applications of CVVH include management of septic shock, clearance of intermediate molecular weight substances, and treatment of fluid overload, thereby improving respiratory function or prognosis in patients with ARDS of various etiologies. This study synthesized existing randomized controlled trials (RCTs) to evaluate whether CVVH offers benefits for ARDS.

## 2 Materials and methods

### 2.1 Search strategy

The final inclusion of 28 randomized controlled trials (RCTs) was conducted by two independent researchers who assessed literature quality using the Cochrane Handbook. The quality evaluation was performed using RevMan 5.3 software, with seven primary assessment criteria: randomization sequence generation, concealment of allocation schemes, blinding of investigators and participants, blinded evaluation of study outcomes, completeness of outcome data, selective reporting of results, and other sources of bias. A literature quality assessment chart was generated. Scoring criteria were as follows: scores of 5 or higher indicated low risk of bias; 3–4 points indicated moderate risk of bias; 1–2 points indicated high risk of bias. In cases of disagreement, a third researcher conducted a consensus decision after discussion (7). Ethical approval was not required because this study only drew from publicly available data.

### 2.2 Selection criteria

#### 2.2.1 Intervention group

The intervention group received CVVH therapy.

#### 2.2.2 Control group

The control group received conventional treatment.

#### 2.2.3 Outcomes

Oxygenation index, mortality rate, ICU length of stay, mechanical ventilation duration, Acute Physiology and Chronic Health Evaluation II score (APACHE II), tumor necrosis factor-alpha (TNF-*α*), ventilator-associated pneumonia (VAP) incidence rate, arterial oxygen partial pressure (PaO₂), arterial carbon dioxide partial pressure (PaCO₂), heart rate (HR), positive end-expiratory pressure (PEEP), interleukin-6 (IL-6), C-reactive protein (CRP), procalcitonin (PCT), white blood cell count (WBC), mean arterial pressure (MAP), cardiac index (CI), and extravascular lung water index (EVLWI).

#### 2.2.4 Types of studies

We included randomized controlled trials (RCTs).

### 2.3 Exclusion criteria

(1) Reviews; (2) Animal studies; (3) Conference papers; (4) Literature with incomplete raw data; (5) Other alternative therapies for ARDS.

### 2.4 Data extraction

The two authors independently extracted data from eligible studies and entered it into a predefined database. We collected potential factors that could lead to significant clinical heterogeneity, including study design, sample size, clinical setting, inclusion and exclusion criteria, CVVH protocols, as well as primary and secondary study endpoints. The primary endpoint of this meta-analysis was mortality rate during the longest follow-up period. Secondary endpoints included oxygenation index, ICU length of stay, and duration of mechanical ventilation.

### 2.5 Risk of bias assessment

The 28 ultimately included studies were all randomized controlled trials. Two researchers independently assessed the quality of these trials using the Cochrane Handbook, employing RevMan 5.3 software for evaluation. Key assessment criteria comprised seven parameters: generation of randomization sequences, concealment of allocation schemes, use of blinding for both investigators and participants, blinded assessment of study outcomes, completeness of outcome data, selective reporting of results, and other sources of bias; a literature quality assessment chart was subsequently created. The scoring criteria were as follows: scores of 5 or higher indicated low risk of bias; 3–4 points indicated moderate risk; 1–2 points indicated high risk. In cases of disagreement regarding the assessment results, a third researcher made the final determination after discussion (8).

### 2.6 Data analysis

To analyze binary outcome indicators, we calculated the odds ratio (OR) and its corresponding 95% confidence interval (CI). To assess study heterogeneity, Cochran’s Q statistic and I^2^ statistic were employed (with I^2^ > 50% as the threshold for significant heterogeneity). Fixed-effect models were used for low statistical inconsistency (I^2^ ≤ 50%), while random-effects models were applied for moderate or high statistical inconsistency (I^2^ > 50%). Publication bias was evaluated using funnel plots of primary outcome measures. For continuous variables of secondary endpoints, standardized mean difference (sMD) and its 95% confidence interval were calculated using random-effects models.

## 3 Results

### 3.1 Literature screening process

**Figure 1:**
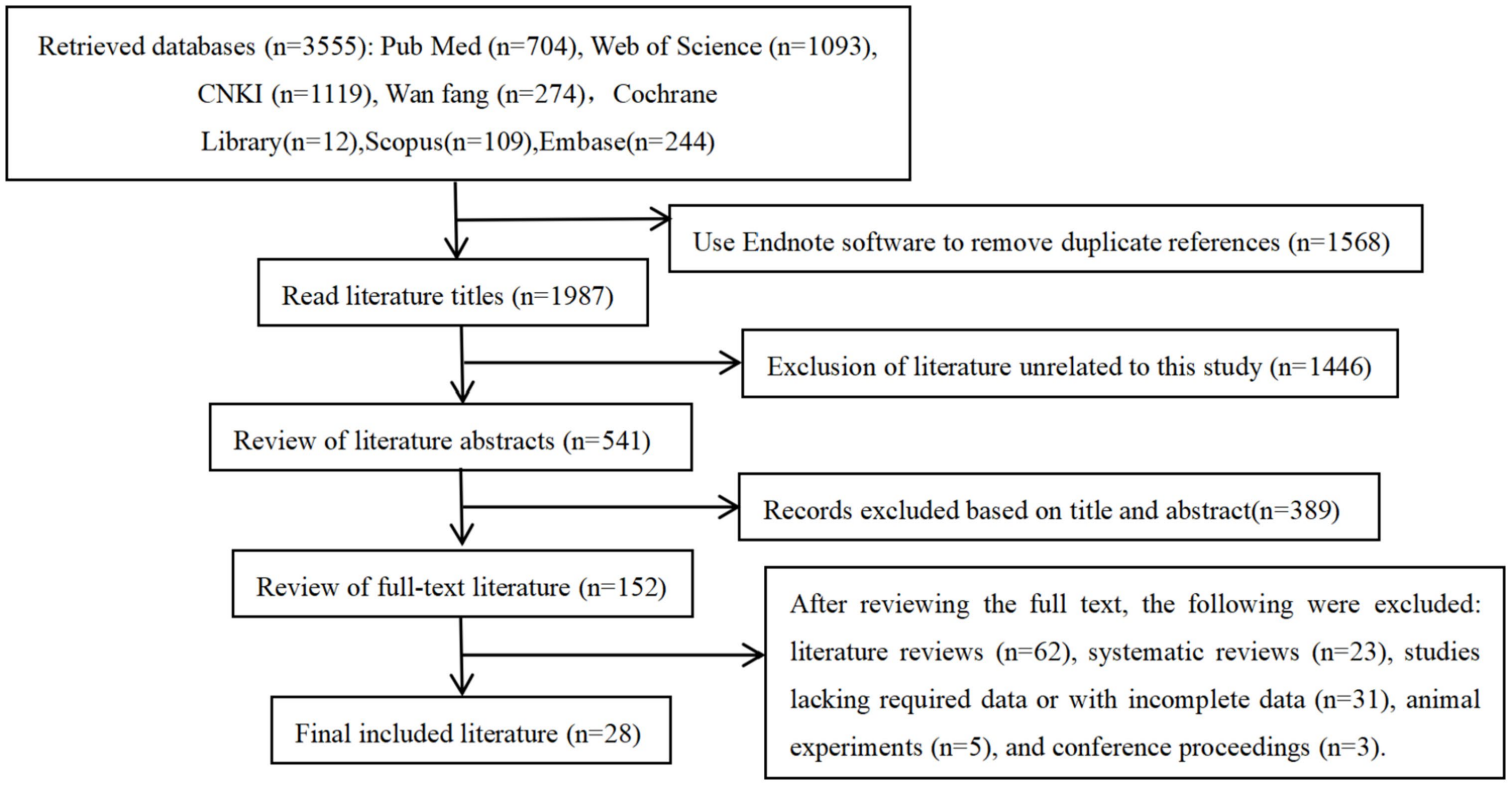
Searching results and study selection and inclusion process.

A total of 3,555 publications were retrieved, including 704 from the PubMed database, 1,093 from the Web of Science database, 1,119 from CNKI, 274 from Wanfang Database, 12 from Cochrane, 244 from Embase, and 109 from Scopus. Ultimately, 28 eligible randomized controlled trials were selected, involving a total of 1739 ARDS patients: 897 cases in the CVVH group and 842 cases in the conventional treatment group. The flowchart for literature screening is shown in Figure 1.

### 3.2 Basic characteristics of the studies

A total of 28 cases were included (9–36). Randomized controlled trials (RCTs) from China compared the efficacy of CVVH versus conventional therapy. The patient age range was approximately 27–76 years (some data presented as mean ± standard deviation or range), with both male and female participants; the total sample size varied from 20 to 149. Outcomes included Mechanical ventilation duration, ICU hospitalization duration, mortality rate, incidence of VAP, oxygenation index, PaO₂, PaCO₂, PEEP, TNF-*α*, IL-6, CRP, PCT, WBC, APACHE II score, MAP, HR, CI, EVLWI (Table 1).

**Table 1:** Basic characteristics of the 28 included studies.

Study	Country	Research type	Age		Gender (male)		Sample size		Intervention study		Outcomes metrics
			CVVH group	Control group	CVVH group	Control group	CVVH group	Control group	CVVH group	Control group	
Meng (36)	China	RCT	62.8 ± 16.4	58.6 ± 17.8	16	17	24	27	CVVH	T	① ② ③ ⑤ ⑦ ⑧ ⑮ ⑯ ⑰ ⑱
Chen et al. (9)	China	RCT	39 ± 14	38 ± 13	35	32	55	50	CVVH	T	① ② ③ ⑤ ⑥ ⑨
Dai et al. (11)	China	RCT	45 ± 6	46 ± 6	14	11	26	20	CVVH	T	① ⑤ ⑥ ⑨ ⑩ ⑪
He et al. (12)	China	RCT	45.1 ± 4.7	46.3 ± 4.4	51	32	89	60	CVVH	T	② ⑨ ⑩
He et al. (13)	China	RCT	43.0 (Overall)	43.0 (Overall)	Ungrouped	Ungrouped	50	50	CVVH	T	③
He et al. (14)	China	RCT	34–57 (range)	34–57 (range)	Ungrouped	Ungrouped	12	12	CVVH	T	⑪ ⑬ ⑭
Hou et al. (16)	China	RCT	48.9 ± 9.2	47.3 ± 8.5	28	25	40	36	CVVH	T	③ ⑤ ⑥ ⑦ ⑨ ⑩ ⑪ ⑭
Huang et al. (17)	China	RCT	44.5 ± 10	45 ± 12	13	12	18	18	CVVH	T	① ③ ④ ⑤ ⑧ ⑱
Jiang et al. (18)	China	RCT	51.9 ± 12.5	52.4 ± 13.8	10	6	17	15	CVVH	T	② ③ ⑤ ⑧ ⑨ ⑩ ⑱
Jin et al. (19)	China	RCT	56 ± 17	53 ± 16	12	14	20	20	CVVH	T	① ② ③ ④ ⑩
Li et al. (20)	China	RCT	57 ± 18	56 ± 17	14	15	25	25	CVVH	T	① ② ③ ④ ⑩
Li et al. (21)	China	RCT	48.2 ± 5.4	52.6 ± 6.3	Ungrouped	Ungrouped	46	43	CVVH	T	⑤ ⑥ ⑦ ⑭
Liu et al. (23)	China	RCT	38 ± 12	38 ± 12	Ungrouped	Ungrouped	19	21	CVVH	T	① ⑤ ⑥ ⑧ ⑯
Shang et al. (24)	China	RCT	39.6	42.8	15	13	23	24	CVVH	T	① ② ③ ④ ⑤ ⑦ ⑨ ⑩ ⑪ ⑭ ⑯
Tang et al. (25)	China	RCT	47.32 ± 5.65	47.36 ± 5.67	20	18	36	36	CVVH	T	② ⑤ ⑥ ⑦ ⑨ ⑩
Wang et al. (26)	China	RCT	60.14 ± 22.16	57.62 ± 21.22	11	13	21	21	CVVH	T	① ② ③ ⑤ ⑧ ⑫ ⑭ ⑱
Xie et al. (27)	China	RCT	27–53 (range)	27–53 (range)	Ungrouped	Ungrouped	21	21	CVVH	T	① ② ③ ④ ⑥ ⑦ ⑮ ⑯
He et al. (15)	China	RCT	49.6 ± 6.7	49.6 ± 6.7	Ungrouped	Ungrouped	40	40	CVVH	T	① ② ③ ⑤ ⑥ ⑪ ⑮
Yue et al. (28)	China	RCT	37.6 ± 3.8	38.1 ± 3.5	21	20	32	32	CVVH	T	① ② ④ ⑤ ⑥ ⑦ ⑭
Zhang et al. (29)	China	RCT	44.0 ± 13.6	41.0 ± 12.3	15	11	37	28	CVVH	T	① ② ③ ⑤ ⑦ ⑮ ⑱
Zhang et al. (30)	China	RCT	56.39 ± 12.24	53.14 ± 15.76	17	14	29	26	CVVH	T	① ② ⑤ ⑨ ⑩ ⑪ ⑫ ⑮ ⑯
Zhang et al. (31)	China	RCT	32–76 (range)	32–76 (range)	Ungrouped	Ungrouped	30	30	CVVH	T	② ③ ⑩
Zhang et al. (32)	China	RCT	46.91 ± 3.67	47.83 ± 4.15	26	27	41	41	CVVH	T	③ ⑨ ⑩ ⑮ ⑯
Zhang et al. (33)	China	RCT	57.12 ± 18.11	56.33 ± 19.44	Ungrouped	Ungrouped	30	30	CVVH	T	③ ⑤ ⑥ ⑦ ⑮ ⑯
Zheng et al. (34)	China	RCT	44.2 ± 10.3 (Overall)	44.2 ± 10.3 (Overall)	Ungrouped	Ungrouped	10	10	CVVH	T	① ② ⑤ ⑨ ⑩ ⑪
Zhou et al. (35)	China	RCT	54 ± 14	53 ± 14	17	19	34	34	CVVH	T	① ② ③ ⑤ ⑨ ⑩ ⑫ ⑬ ⑰
Liang et al. (22)	China	RCT	54.61 ± 9.84	52.33 ± 10.27	20	18	30	30	CVVH	T	① ② ③ ⑤ ⑥ ⑦ ⑨ ⑩ ⑪ ⑫ ⑭
Cheng et al. (10)	China	RCT	44.61 ± 3.54	44.56 ± 3.58	23	24	42	42	CVVH	T	⑤ ⑥ ⑦ ⑨ ⑩ ⑪ ⑭

RCT, randomized controlled trial; T, conventional treatment; Outcome indicators: ① mechanical ventilation duration; ② ICU hospitalization duration; ③ mortality rate; ④ incidence of VAP; ⑤ oxygenation index; ⑥ PaO₂; ⑦ PaCO₂; ⑧ PEEP; ⑨ TNF-α; ⑩ IL-6; ⑪ CRP; ⑫ PCT; ⑬ WBC; ⑭ APACHE II score; ⑮ MAP; ⑯ HR; ⑰ CI; ⑱ EVLWI.

### 3.3 Risk of bias in included studies

**Figure 2:**
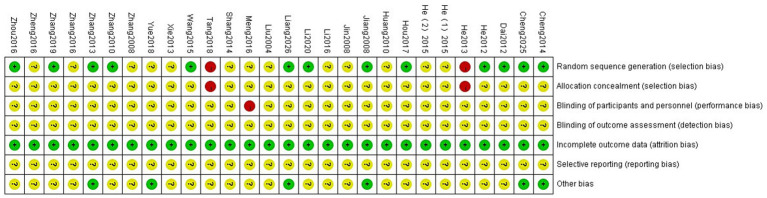
Overall quality assessment of included literature. “+” indicates low risk, “?” indicates unknown risk, and “−” indicates high risk.

**Figure 3:**
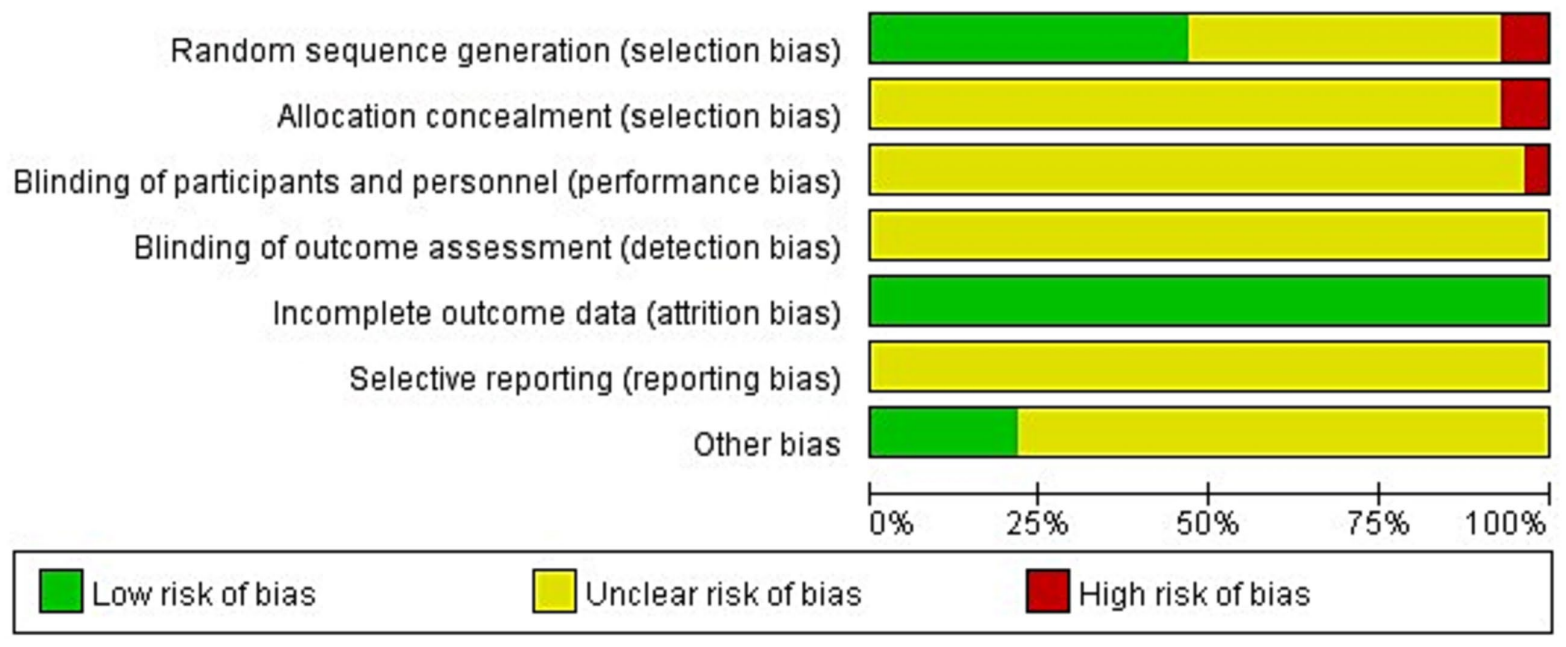
Percentage statistical chart of various items in literature quality assessment. Low risk of bias indicates low bias risk, unclear risk of bias indicates unknown risk, high risk of bias indicates high bias risk.

The final inclusion of 28 randomized controlled trials underwent quality assessment using RevMan 5.3 software. As shown in Figure 2, all studies achieved scores of 5 or higher, indicating low bias risk. Figure 3 reveals that 100% of studies had low risk for selective reporting, incomplete outcome data, random sequence generation, and other bias sources. More than 75% of studies also showed low risk for allocation concealment and blinding of participants and personnel, as well as blinding of outcome assessment. Overall, the included studies demonstrated high methodological quality.

### 3.4 Meta-analysis results

#### 3.4.1 Primary outcomes measures

##### 3.4.1.1 Mortality rate

**Figure 4:**
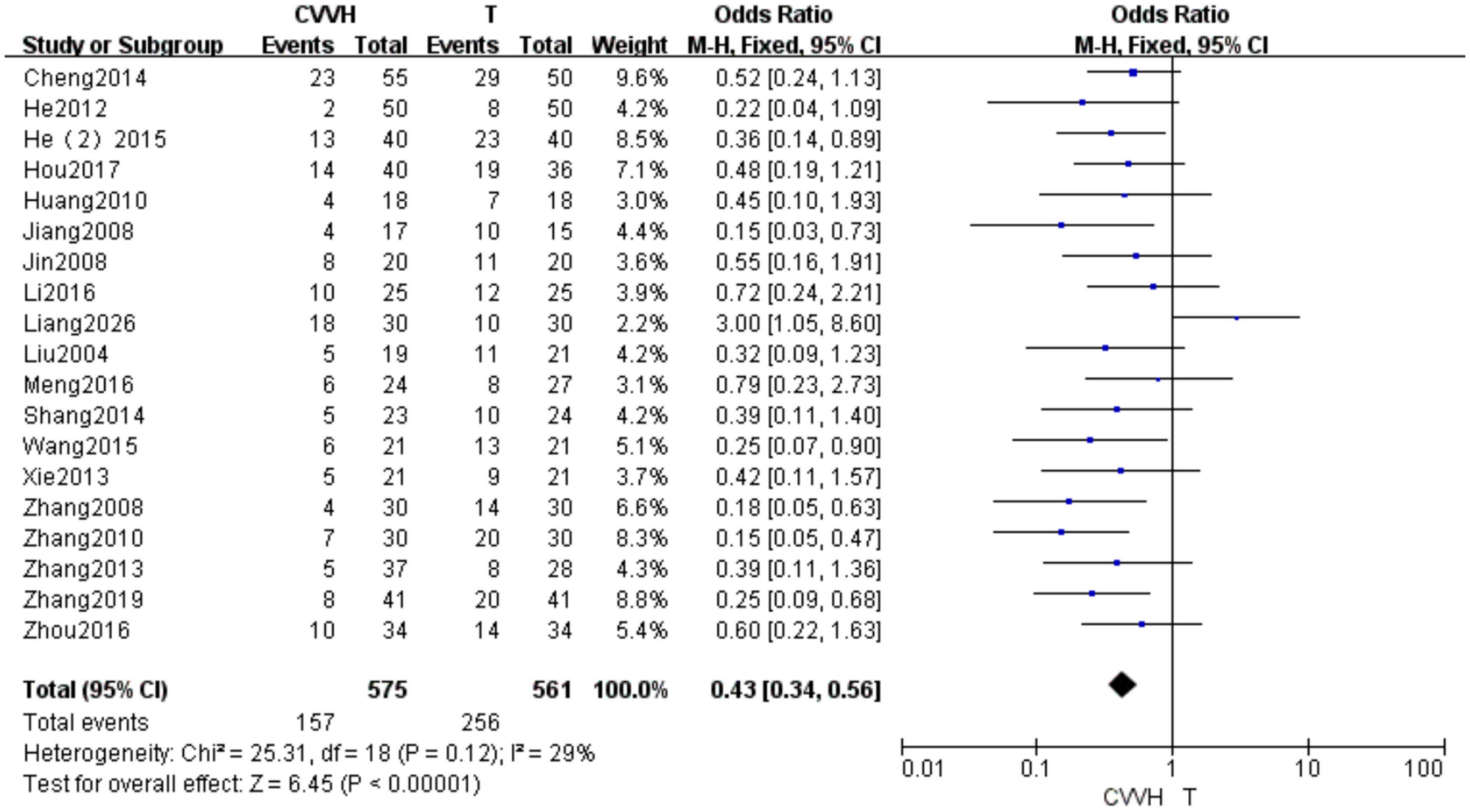
Forest plot of mortality rate in the CVVH group vs. T group.

Nineteen studies reported mortality rates (9, 12, 15, 18, 20, 22, 24–27, 29–31, 34–36). One study attributed the cause of death exclusively to pancreatitis, another solely to sepsis, while the remaining seventeen studies listed multiple critical conditions as causes of death. None of the included studies explicitly reported whether acute kidney injury (AKI) was present. Meta-analysis results showed no statistical heterogeneity among studies (*p* = 0.12 I^2^ = 29%), thus a fixed-effects model was adopted. Subgroup analysis was not performed due to low heterogeneity among studies and insufficient information on grouping variables. A statistically significant difference in mortality rates was observed between the CVVH group and the conventional treatment group post-treatment (OR = 0.43, 95% CI (0.34, 0.56), *p*<0.00001), as illustrated in Figure 4.

#### 3.4.2 Secondary outcomes

The secondary outcome endpoints comprised 17 parameters, including VAP incidence rate, ICU length of stay, mechanical ventilation duration, oxygenation index, PaO₂, PaCO₂, PEEP, TNF-*α*, IL-6, CRP, PCT, WBC, APACHE II score, mean arterial pressure (MAP), heart rate (HR), cardiac index (CI), and EVLWI.

##### 3.4.2.1 Mechanical ventilation duration

**Figure 5:**
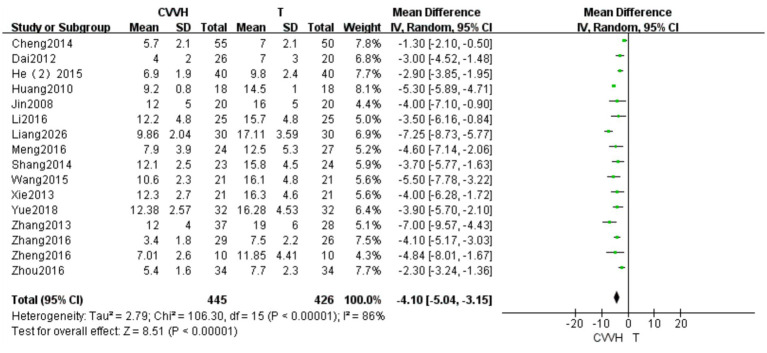
Forest plot of mechanical ventilation duration in the CVVH group vs. T group.

Sixteen randomized controlled trials documented the mechanical ventilation duration in both treatment groups during therapy (9, 11, 15, 17, 19, 20, 22–24, 26–30, 34–36). Meta-analysis revealed statistical heterogeneity among studies (*p*<0.00001, I^2^ = 86%), necessitating the use of a random effects model. Post-treatment mechanical ventilation duration showed statistically significant differences between the CVVH group and conventional treatment group (MD = −4.10, 95% CI (−5.04, −3.15), *p*<0.00001), as illustrated in Figure 5.

The Egger test suggested a potential publication bias (*p* = 0.006). After trim-and-fill adjustment, the pooled MD for mechanical ventilation duration was −3.62 (95% CI, −4.58 to −2.66), suggesting that the previously observed effect might be slightly overestimated due to publication bias.

##### 3.4.2.2 ICU hospitalization duration

**Figure 6:**
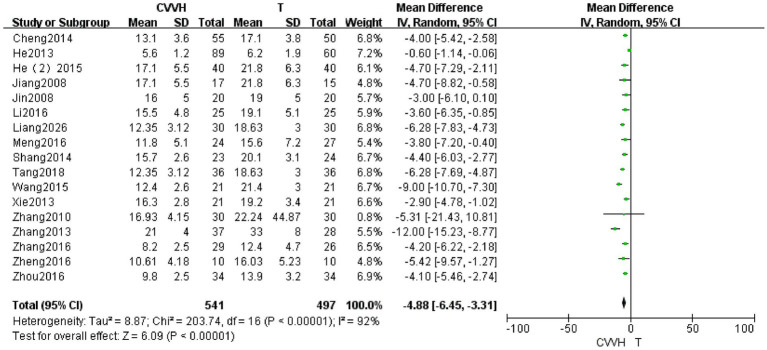
Forest plot of ICU hospitalization duration in the CVVH group vs. T group.

Seventeen randomized controlled trials documented the ICU hospitalization duration between the two treatment groups during the therapeutic process (9, 12, 15, 18, 20, 22, 24–27, 29–31, 34–36). Meta-analysis results indicated statistically significant heterogeneity among studies (*p* < 0.00001, I^2^ = 92%). Therefore, a random effects was adopted. Post-treatment ICU hospitalization durations showed statistically significant differences between the CVVH group and conventional treatment group (MD = −4.88, 95% CI (−6.45, −3.31), *p*<0.00001), as shown in Figure 6.

Egger’s test suggested the presence of small-study effects (*p* = 0.006). The trim-and-fill analysis imputed no missing studies, and the adjusted effect estimate (MD = −4.88, 95% CI: −6.45 to −3.31) was identical to the original pooled result, indicating that the conclusion is robust despite the presence of small-study effects.

##### 3.4.2.3 Incidence of VAP

**Figure 7:**
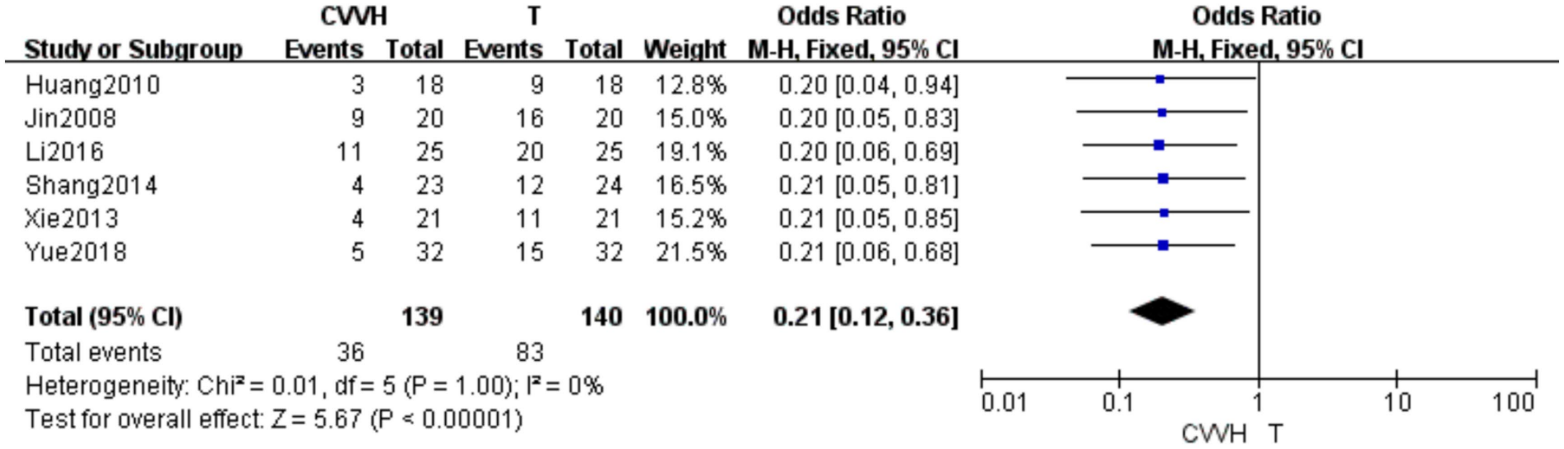
Forest plot of incidence of VAP in the CVVH group vs. T group.

Six randomized controlled trials documented the incidence of VAP in both groups during treatment (17, 19, 20, 24, 27, 28). Meta-analysis results showed no statistical heterogeneity among studies (*p* = 1.00, I^2^ = 0%), thus a fixed-effects model was adopted. The incidence of VAP in the CVVH group and conventional treatment group after treatment was statistically significant (OR = 0.21, 95% CI (0.12, 0.36), *p*<0.00001), as illustrated in Figure 7.

##### 3.4.2.4 Oxygenation index

**Figure 8:**
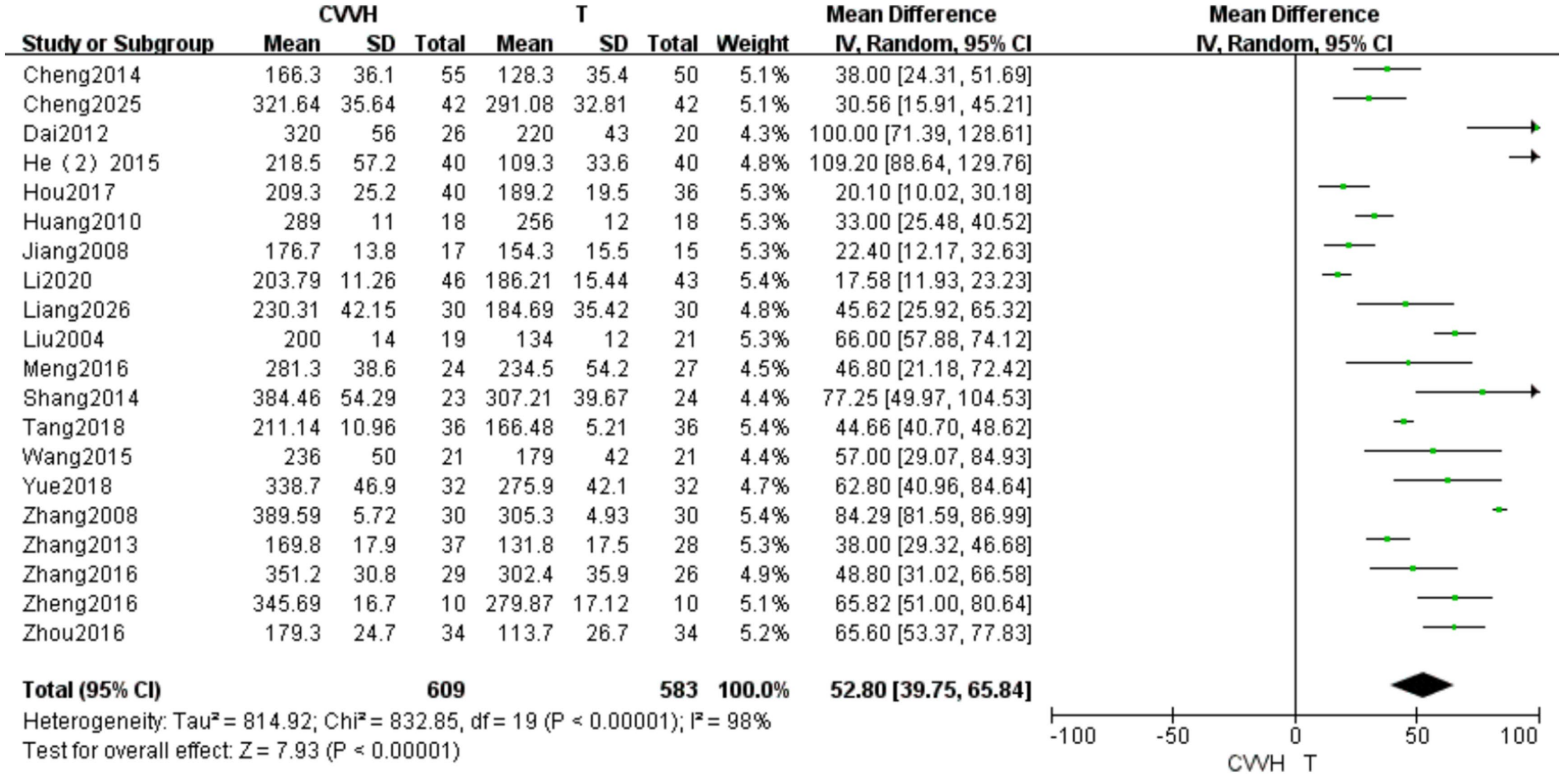
Forest plot of oxygenation indice in the CVVH group vs. T group.

Twenty randomized controlled trials documented oxygenation index data during treatment (9–11, 15–18, 21–26, 28–30, 33, 35, 36). Meta-analysis results indicated high statistical heterogeneity among studies (*p* < 0.00001, I^2^ = 98%), thus a random effects model was adopted. A statistically significant difference in oxygenation index was observed between the CVVH group and conventional treatment group post-treatment (MD = 52.80, 95% CI (39.75, 65.84), *p* < 0.00001), as shown in Figure 8.

The Egger test revealed significant publication bias (*p* < 0.001). Despite significant publication bias detected by the Egger test (*p* < 0.001), trim-and-fill adjustment yielded a pooled MD of 38.50 (95% CI: 24.10 to 52.90, *p* < 0.01) with five imputed studies. The attenuated but still significant effect confirms that CVVH improves oxygenation index, though the magnitude may be overestimated in small-sample studies.

##### 3.4.2.5 PaO₂

**Figure 9:**
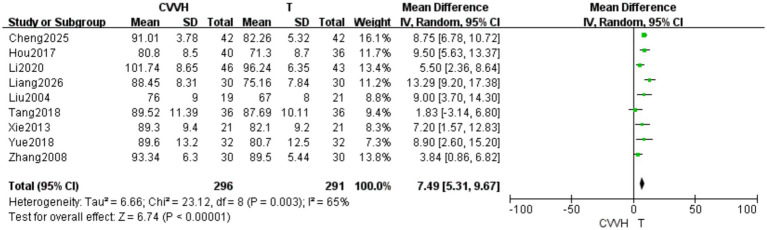
Forest plot of PaO_2_ in the CVVH group vs. T group.

Nine randomized controlled trials recorded PaO_2_ levels in both groups during treatment (9, 16, 21–23, 25, 27, 28, 33). Meta-analysis revealed statistically significant heterogeneity among studies (*p* = 0.003, I^2^ = 65%), necessitating the use of a random effects model. Significant differences in PaO₂ levels were observed between the CVVH group and the conventional treatment group. (MD = 7.49, 95% CI (5.31, 9.67), *p*<0.00001), as shown in Figure 9.

##### 3.4.2.6 PaCO₂

**Figure 10:**
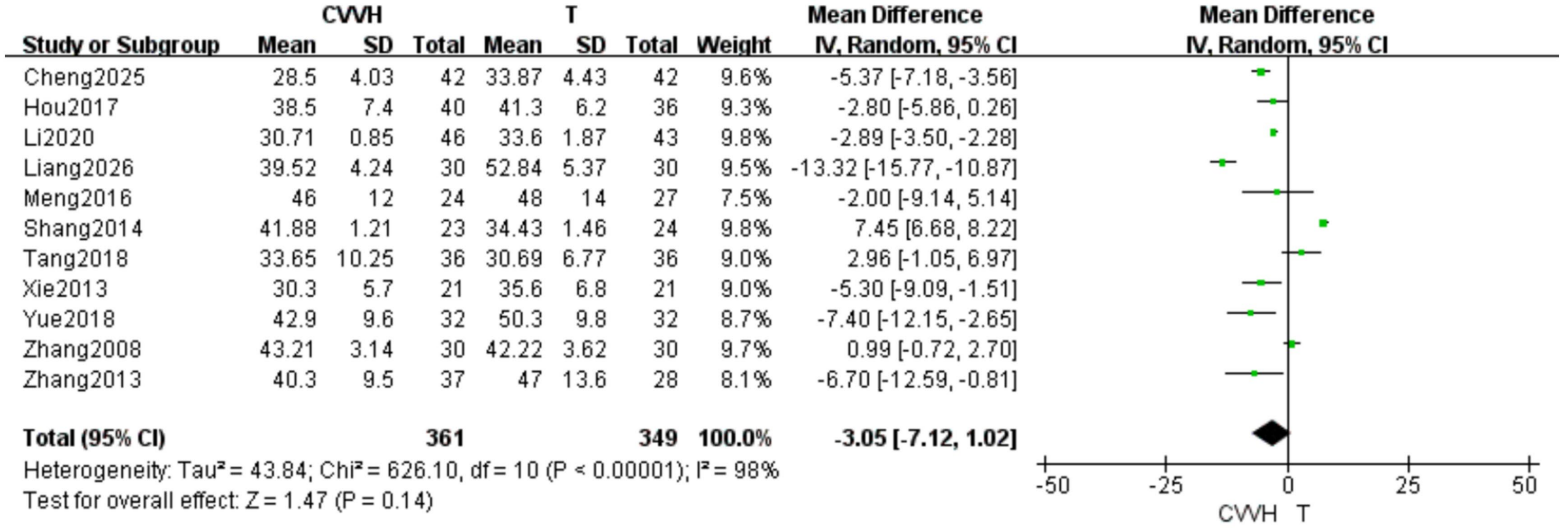
Forest plot of PaCO_2_ in the CVVH group vs. T group.

Eleven randomized controlled trials recorded PaCO_2_ levels in both groups during treatment (9, 16, 21, 22, 24, 25, 27–29, 33, 36). Meta-analysis revealed statistically significant heterogeneity among studies (*p* < 0.00001, I^2^ = 98%), necessitating the use of a random effects model. No significant difference in PaCO_2_ between the CVVH group and conventional treatment group was observed post-treatment (MD = −3.05, 95% CI (−7.12, 1.02), *p* = 0.14), as shown in Figure 10.

##### 3.4.2.7 PEEP

**Figure 11:**
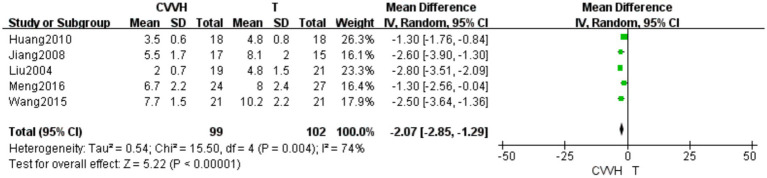
Forest plot of PEEP in the CVVH group vs. T group.

Five randomized controlled trials documented PEEP in both treatment groups during therapy (17, 18, 23, 26, 36). Meta-analysis revealed statistical heterogeneity among studies (*p* = 0.004, I^2^ = 74%), necessitating the use of a random effects model. Significant differences in PEEP were observed between the CVVH group and the conventional treatment group. (MD = −2.07, 95% CI (−2.85, −1.29), *p*<0.00001), as illustrated in Figure 11.

Despite evidence of publication bias (Egger test, *p* = 0.021), trim-and-fill adjustment yielded a pooled MD of −1.85 (95% CI: −2.68 to −1.02, *p* < 0.01) with one imputed study, confirming that CVVH significantly reduces PEEP in ARDS patients.

##### 3.4.2.8 TNF-*α*

**Figure 12:**
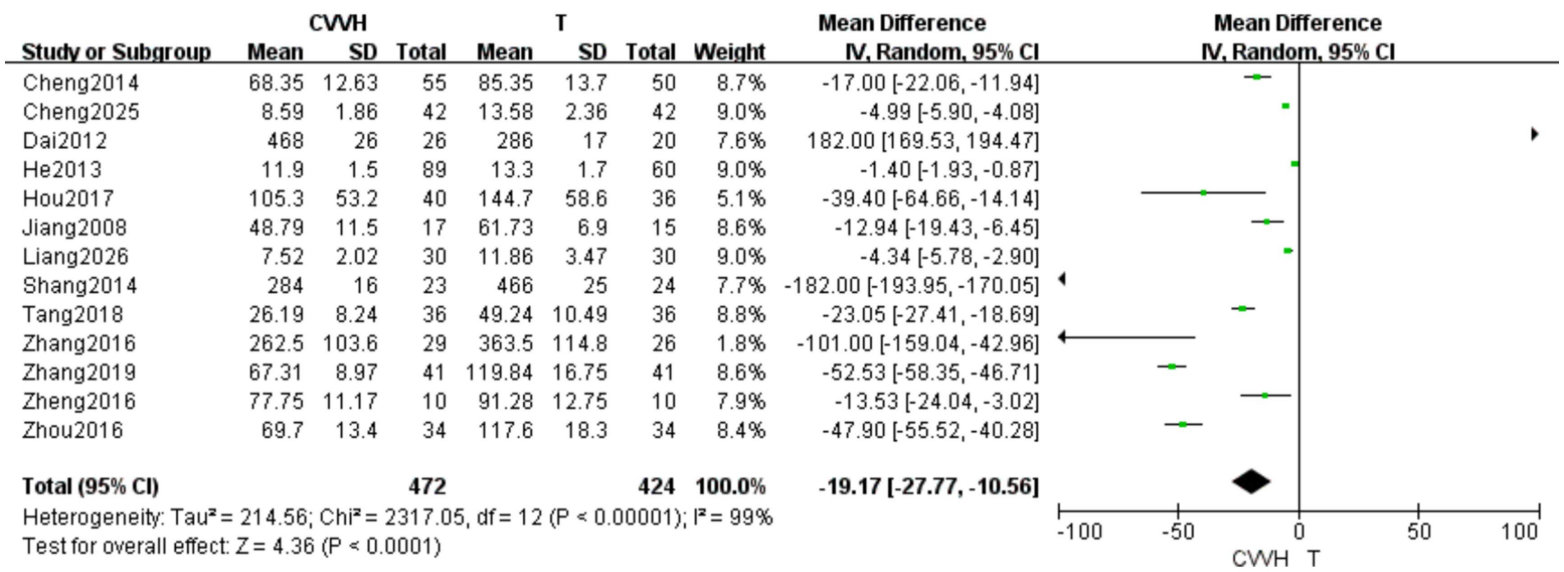
Forest plot of TNF-*α* in the CVVH group vs. T group.

Thirteen randomized controlled trials documented the TNF-α levels in both groups during treatment (9–12, 16, 18, 22, 24, 25, 30, 32, 34, 35). Meta-analysis revealed statistically significant heterogeneity among studies (*p* < 0.00001, I^2^ = 99%), necessitating the use of a random effects model. There was a significant difference in TNF-α levels between the CVVH group and the conventional treatment group. (MD = −19.17, 95% CI (−27.77, −10.56), *p*<0.0001), as shown in Figure 12.

##### 3.4.2.9 IL-6

**Figure 13:**
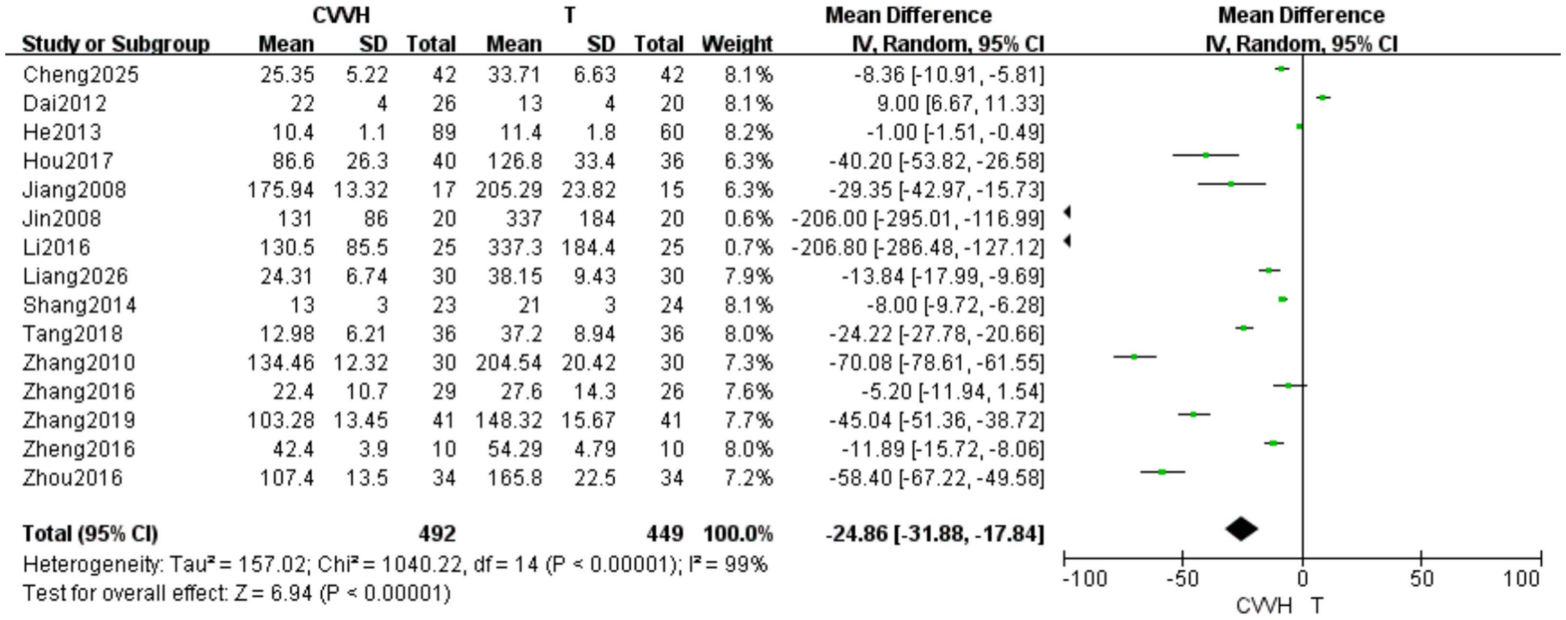
Forest plot of IL-6 in the CVVH group vs. T group.

Fifteen randomized controlled trials documented the IL-6 levels in both treatment groups during therapy (9, 11, 12, 16, 18–20, 22, 24, 25, 30–32, 34, 35). Meta-analysis revealed statistical heterogeneity among studies (*p*<0.00001, I^2^ = 99%), necessitating the use of a random effects model. Significant differences in IL-6 levels were observed between the CVVH group and the conventional treatment group. (MD = −24.86, 95% CI (−31.88, −17.84), *p*<0.00001), as illustrated in Figure 13.

##### 3.4.2.10 CRP

**Figure 14:**
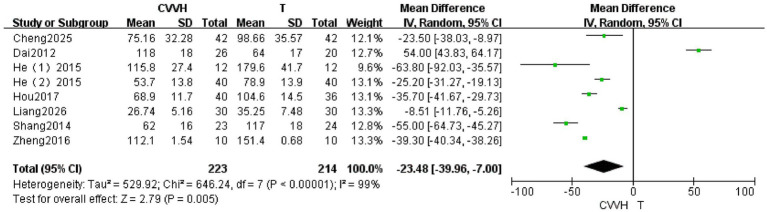
Forest plot of *CRP* in the CVVH group vs. T group.

Eight randomized controlled trials documented the CRP levels in both treatment groups during therapy (10, 11, 14–16, 22, 24, 34). Meta-analysis revealed statistical heterogeneity among studies (*p*<0.00001, I^2^ = 99%), necessitating the use of a random effects model. Significant differences in CRP levels were observed between the CVVH group and the conventional treatment group. (MD = −23.48, 95% CI (−39.96, −7.00), *p* = 0.005), as illustrated in Figure 14.

##### 3.4.2.11 PCT

**Figure 15:**
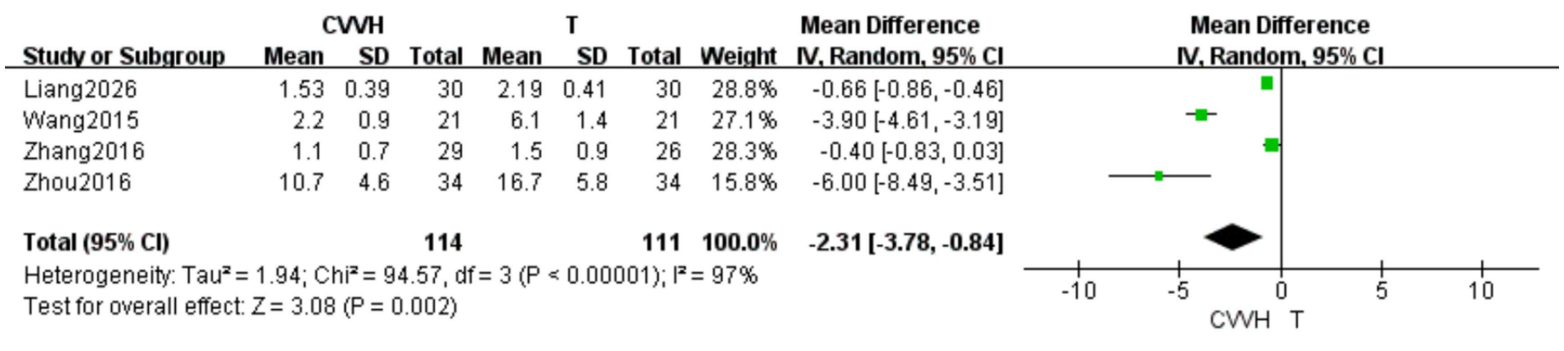
Forest plot of PCT in the CVVH group vs. T group.

Four randomized controlled trials documented the PCT levels in both treatment groups during therapy (22, 26, 30, 35). Meta-analysis revealed statistical heterogeneity among studies (*p*<0.00001, I^2^ = 97%), necessitating the use of a random effects model. Significant differences in PCT levels were observed between the CVVH group and the conventional treatment group. (MD = −2.31, 95% CI (−3.78, −0.84), *p* = 0.002), as illustrated in Figure 15.

##### 3.4.2.12 WBC

**Figure 16:**
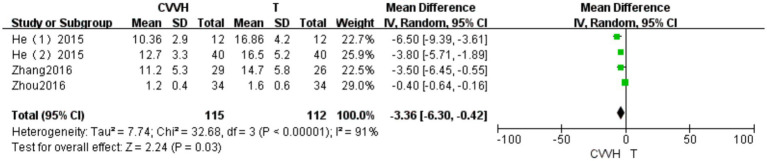
Forest plot of WBC in the CVVH group vs. T group.

Four randomized controlled trials documented the WBC levels in both treatment groups during therapy (14, 15, 30, 35). Meta-analysis revealed statistical heterogeneity among studies (*p*<0.00001, I^2^ = 91%), necessitating the use of a random effects model. Significant differences in WBC levels were observed between the CVVH group and the conventional treatment group. (MD = −3.36, 95% CI (−6.30, −0.42), *p* = 0.03), as illustrated in Figure 16.

##### 3.4.2.13 APACHE II

**Figure 17:**
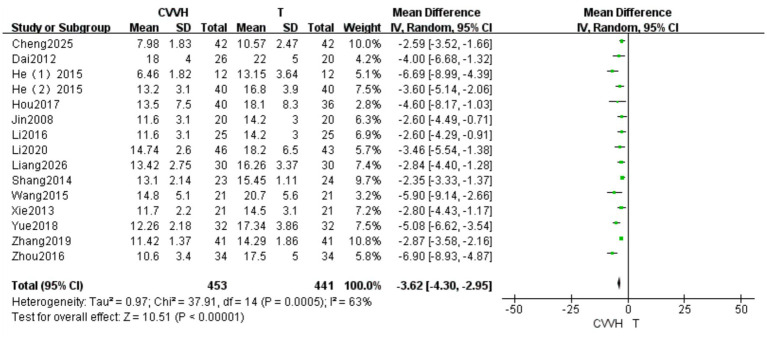
Forest plot of APACHE II in the CVVH group vs. T group.

Fifteen randomized controlled trials documented the APACHE II scores between the two groups during treatment (10, 11, 14–16, 19–22, 24, 26–28, 32, 35). Meta-analysis revealed statistical heterogeneity among studies (*p* = 0.00005, I^2^ = 63%), thus a random effects model was adopted. Statistical differences in APACHE II scores between the CVVH group and conventional treatment group were observed post-treatment (MD = −3.62, 95% CI (−4.30, −2.95), *p* < 0.00001), as illustrated in Figure 17.

##### 3.4.2.14 MAP

**Figure 18:**
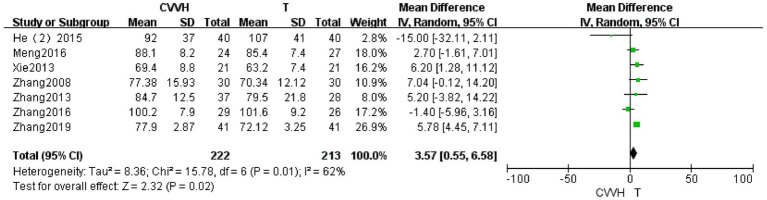
Forest plot of MAP in the CVVH group vs. T group.

Seven randomized controlled trials documented the MAP levels between the two groups during treatment (15, 27, 29, 30, 32, 33, 36). The meta-analysis results demonstrated statistical heterogeneity among the studies (*p* = 0.01, I^2^ = 62%), thus a random effects model was adopted. Statistical differences in MAP between the CVVH group and conventional treatment group were observed post-treatment (MD = 3.57, 95% CI (0.55, 6.58), *p* = 0.02), as illustrated in Figure 18.

##### 3.4.2.15 HR

**Figure 19:**
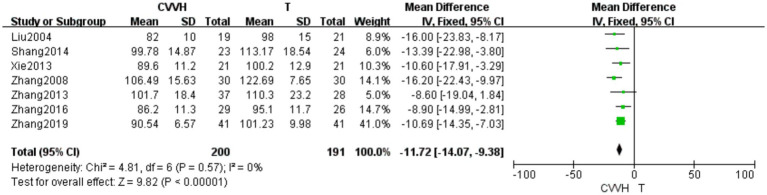
Forest plot of HR in the CVVH group vs. T group.

A total of seven randomized controlled trials documented the HR between the two groups during treatment (23, 24, 27, 29, 30, 32, 33). Meta-analysis results showed no statistical heterogeneity among studies (*p* = 0.57, I^2^ = 0%), thus a fixed-effects model was adopted. The HR between the CVVH group and the conventional treatment group after treatment was statistically significant (MD = −11.72, 95% CI (−14.07, −9.38), *p* < 0.00001), as illustrated in Figure 19.

##### 3.4.2.16 CI

**Figure 20:**
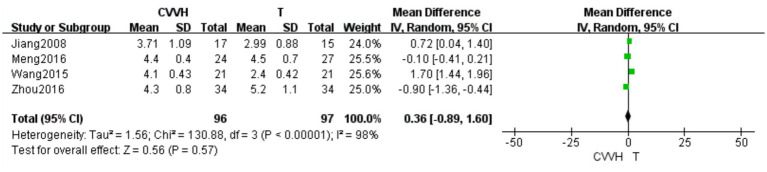
Forest plot of CI in the CVVH group vs. T group.

Four randomized controlled trials documented the CI levels between the two groups during treatment (18, 26, 35, 36). The meta-analysis results demonstrated statistical heterogeneity among the studies (*p*<0.00001, I^2^ = 98%), thus a random effects model was adopted. No statistically significant difference in the CI was observed between the CVVH group and the conventional treatment group after therapy (MD = 0.36, 95% CI (−0.89, 1.60), *p* = 0.57), as illustrated in Figure 20.

Despite evidence of small-study effects (Egger test, *p* = 0.011), trim-and-fill analysis imputed no missing studies, and the adjusted effect estimate (MD = 0.36, 95% CI: −0.89 to 1.60, *p* = 0.57) was identical to the original result, confirming the absence of a significant difference in cardiac index.

##### 3.4.2.17 EVLWI

**Figure 21:**
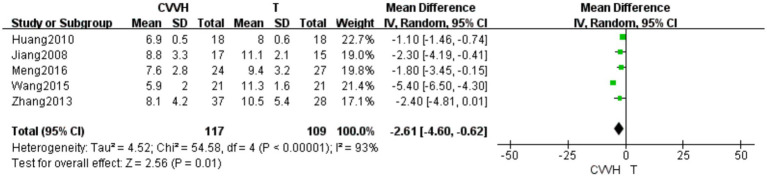
Forest plot of EVLWI in the CVVH group vs. T group.

Five randomized controlled trials documented the EVLWI levels between the two groups during treatment (17, 18, 26, 29, 36). The meta-analysis results demonstrated statistical heterogeneity among the studies (*p*<0.00001, I^2^ = 93%), thus a random effects model was adopted. Statistical differences in EVLWI between the CVVH group and conventional treatment group were observed post-treatment (MD = −2.61, 95% CI (−4.60, −0.62), *p* < 0.00001), as illustrated in Figure 21.

The Egger test suggested potential publication bias (*p* = 0.039). After applying the trim-and-fill method, one missing study was imputed, and the adjusted pooled MD for EVLWI was −2.15 (95% CI, −4.20 to −0.10, *p* < 0.05). Although the effect size was attenuated compared to the original estimate (MD = −2.61), the result remained statistically significant, supporting the conclusion that CVVH reduces EVLWI in ARDS patients. However, due to the limited number of studies (*n* = 5) and high heterogeneity (I^2^ = 93%), these findings should be interpreted with caution.

#### 3.4.3 Sensitivity analysis

After systematically excluding each study individually for every outcome endpoint, no significant changes were observed in the results. The statistical significance of the findings remained intact, highlighting the sustained stability and robustness of this meta-analysis. Detailed data on all outcome endpoints and their statistical analyses are presented in Table 2.

**Table 2:** The summary of outcome indicators.

Outcomes Indicator	Initial data analysis							Egger test *p*-value	Publication bias	After modification using the trim-and-fill method				sensitivity analysis
	Number of Studies	OR/MD	95% CI	I^2^	model	*p* value	Statistically significant			OR/MD	95% CI	*p*	robust	
Death rate	19	OR = 0.43	(0.34, 0.56)	29%	Fixed	<0.00001	√	0.302	NA	NA	NA	<0.01	√	√
VAP incidence	6	OR = 0.21	(0.12, 0.36)	0%	Fixed	<0.00001	√	0.152	NA	NA	NA	NA	√	√
ICU hospitalization duration (days)	17	MD = −4.88	(−6.45, −3.31)	92%	Random	<0.00001	√	0.006	Y	-4.88	(−6.45, 3.31)	NA	√	√
Mechanical ventilation duration (days)	16	MD = −4.10	(−5.04, −3.15)	90%	Random	<0.00001	√	0.006	Y	-3.62	(−4.58, −2.66)	NA	√	√
oxygenation index	20	MD = 52.80	(39.75, 65.84)	98%	Random	<0.00001	√	0.000	Y	38.5	(24.10, 52.90)	NA	√	√
PaO₂	9	MD = 7.49	(5.31, 9.67)	65%	Random	<0.00001	√	0.349	NA	NA	NA	NA	√	√
PaCO₂	11	MD = −3.05	(−7.12, 1.02)	98%	Random	0.14	×	0.807	NA	NA	NA	NA	√	√
PEEP	5	MD = −2.07	(−2.85, −1.29)	74%	Random	<0.00001	√	0.021	Y	NA	NA	NA	√	√
TNF-α	13	MD = −19.17	(−27.77, −10.56)	99%	Random	<0.0001	√	0.624	NA	NA	NA	NA	√	√
IL-6	15	MD = −24.86	(−31.88, −17.84)	99%	Random	<0.00001	√	0.115	NA	NA	NA	NA	√	√
CRP	8	MD = −23.48	(−39.96, −7.00)	99%	Random	0.005	√	0.425	NA	NA	NA	NA	√	√
PCT	4	MD = −2.31	(−3.78, −0.84)	97%	Random	0.002	√	0.113	NA	NA	NA	NA	√	√
WBC	4	MD = −3.36	(−6.30, −0.42)	91%	Random	0.03	√	0.157	NA	NA	NA	NA	√	√
APACHE II	15	MD = −3.62	(−4.30, −2.95)	63%	Random	<0.00001	√	0.089	NA	NA	NA	NA	√	√
MAP	7	MD = 3.57	(0.55, 6.58)	62%	Random	0.02	√	0.340	NA	NA	NA	NA	√	√
HR	7	MD = −11.72	(−14.07, −9.38)	0%	Fixed	<0.00001	√	0.761	NA	NA	NA	NA	√	√
CI	4	MD = 0.36	(−0.89, 1.60)	98%	Random	0.57	×	0.011	Y	0.36	(−0.89, 1.60)	NA	√	√
EVLWI	5	MD = −2.61	(−4.60, −0.62)	93%	Random	<0.00001	√	0.039	Y	2.15	(−4.20, −0.10)	NA	√	√

√ means yes. × means no. Y means publication bias.

## 4 Discussion

The etiology of ARDS includes both pulmonary and extrapulmonary factors. Pulmonary factors primarily refer to pneumonia and chest trauma, whereas extrapulmonary factors encompass sepsis, pancreatitis, trauma, burns, shock, multiple blood transfusions, aspiration, medications, toxins, and other conditions that trigger inflammatory responses (3, 37, 38). These reactions activate effector cells, leading to the release of large amounts of cytokines and inflammatory mediators. This series of events ultimately results in a “cytokine storm,” triggering a cascade reaction that culminates in acute respiratory distress syndrome (ARDS) (3). Therefore, timely removal of circulating inflammatory mediators and inhibition of the inflammatory response pathway are critical measures in the treatment of ARDS. Currently, there is no specific therapeutic approach for ARDS; mechanical ventilation serves as the cornerstone of management. Although advancements in ARDS treatment have significantly improved prognosis, mortality rates remain elevated (3). Continuous venous venous hemofiltration (CVVH) has been widely adopted as a renal support modality for critically ill patients in intensive care units (ICUs) and demonstrates significant therapeutic efficacy. Its application in acute respiratory distress syndrome (ARDS) patients has been extensively investigated. CVVH removes certain inflammatory mediators through convection and/or adsorption, potentially exerting beneficial regulatory effects on immune responses and thereby reducing both the incidence and mortality rates of ARDS—even in patients without acute kidney injury who require renal replacement therapy (RRT) (39, 40). Therefore, the conclusions of this study should be limited to the CVVH modality and should not be directly generalized to other CRRT methods. The article examines the characteristics of CVVH versus conventional therapy in terms of oxygenation index, mortality rate, ICU hospitalization duration, mechanical ventilation time, APACHE II score, TNF-*α* levels, VAP incidence, PaO₂, PaCO₂, and heart rate in ARDS patients. A total of 28 randomized controlled trials were included, most of which are medium-to-high-quality studies.

The meta-analysis results demonstrated that, compared to the control group, the application of continuous venous vitamin infusion (CVVH) therapy significantly reduced serum levels of TNF-*α*, IL-6, CRP, PCT, and WBC in patients with acute respiratory distress syndrome (ARDS). These findings suggest that CVVH may exert significant anti-inflammatory effects in the treatment of ARDS. During the onset and progression of acute respiratory distress syndrome (ARDS), most underlying diseases and precipitating factors do not directly affect the lungs; rather, systemic inflammatory responses play a predominant role in the development of ARDS, specifically the “cytokine storm,” which is primarily driven by the excessive release of pro-inflammatory cytokines such as TNF-α and IL-6, as well as acute-phase response proteins like CRP and PCT. Among these biomarkers, TNF-α is recognized as one of the most critical cytokines involved in ARDS pathogenesis. TNF-α activates damaged platelets, granulocytes, and skin cells, triggering a cascading waterfall-like chain reaction that induces tissue cell damage. IL-6, produced by both lymphatic and non-lymphatic tissue cells, stimulates hepatocytes to produce acute-phase response protein (CRP), thereby participating in the inflammatory mediator cascade. These findings demonstrate that CVVH can eliminate substantial amounts of inflammatory mediators, suppress inflammatory responses, and restore the body’s immune homeostasis (25). The reduction in CRP and PCT levels (commonly used clinical biomarkers for infection/inflammation) further supports the alleviation of systemic inflammation. Conversely, a decrease in white blood cell (WBC) count may reflect diminished inflammatory stimulation of the bone marrow or better control of underlying infection/sepsis; however, this finding should be interpreted with caution due to potential confounding effects from concomitant therapies such as antibiotics or glucocorticoids.

ARDS is characterized by non-cardiogenic pulmonary edema resulting from the accumulation of extravascular lung water (EVLW). EVLW reflects pulmonary edema, serves as a prognostic factor for ARDS patients, correlates with disease severity, and constitutes a significant contributor to refractory hypoxemia in ARDS patients. The volume of EVLW typically ranges between 3 and 7 mL/kg; levels exceeding 10 mL/kg are associated with clinical pulmonary edema (36). During ARDS progression, increased extravascular lung water leads to marked declines in pulmonary oxygenation index and static compliance. Extravascular lung water represents the sole bedside parameter capable of quantitatively assessing the extent of pulmonary capillary damage and its permeability, playing a critical role in fluid management of ARDS and serving as an independent risk factor for predicting disease severity and prognosis in critically ill patients. High-volume hemofiltration reduces pulmonary vascular permeability in patients with septic shock, decreases extravascular lung water, enhances alveolar-arterial oxygen exchange, and improves prognosis. The mechanism by which continuous venous hemoperfusion (CVVH) removes extravascular lung water may involve the following process: during blood flow through the filter, water is expelled at a predetermined rate; plasma protein concentration and osmotic pressure significantly increase upon return to the body; interstitial fluid and alveolar water then migrate into alveolar capillaries due to hydrostatic pressure when passing through the lungs, where they are subsequently re-excreted via filtration, maintaining a dynamic equilibrium that effectively eliminates substantial extravascular lung water, corrects interstitial and alveolar edema, reduces airway pressure, and improves gas exchange and tissue oxygenation (26).

The improvement in oxygenation parameters may be attributed to CVVH’s ability to rapidly remove excess heat from ARDS patients, thereby reducing the basal metabolic rate and decreasing oxygen consumption, which subsequently enhances the oxygenation index (3). However, the Egger test revealed significant publication bias for the oxygenation index (*p* < 0.001), with a positive intercept, suggesting that small-sample studies may have overestimated the efficacy of CVVH. Therefore, this conclusion should be interpreted with caution and requires further validation through larger-scale, high-quality studies. Additionally, the lack of significant impact of CVVH on PaCO₂ warrants further discussion. PaCO₂ is primarily determined by alveolar ventilation rather than oxygenation or lung water content; unless combined with respiratory support modulation or an extracorporeal CO₂ removal device, CVVH does not directly enhance CO₂ clearance. The absence of significant differences in PaCO₂ between the two groups suggests that CVVH’s beneficial effects on gas exchange are primarily oxygen-related, likely reflecting improvements in the ventilation/perfusion ratio rather than changes in alveolar ventilation itself.

The determination of the optimal PEEP level in the treatment of ARDS patients has consistently been a major concern for clinicians. Although high PEE*p* values are often required to prevent alveolar collapse, excessively high PEEP can easily lead to barotrauma or even pneumothorax (26). Our study results indicate that compared with the conventional treatment group, patients treated with CVVH required lower ventilator PEEP values, resulting in improved oxygenation indices, reduced long-term mortality, and favorable prognostic outcomes.

The APACHE II score is an internationally recognized critical condition scoring system associated with mortality rates in ICU patients. The results of this study demonstrated that patients receiving continuous venous ventilation (CVVH) exhibited significantly lower APACHE II scores and mortality rates compared to those receiving conventional treatment, indicating improved overall condition, disease severity, and prognosis among CVVH-treated patients (32).

ARDS patients receiving CVVH therapy exhibited significantly shorter mechanical ventilation durations and a relatively lower incidence of VAP compared to those receiving conventional treatment alone. This may be attributed to CVVH’s efficacy in reducing EVLWI, rapidly improving oxygenation, and lowering PEEP thereby mitigating VAP; additionally, it may also be associated with CVVH’s ability to eliminate inflammatory cytokines from the body (10).

Heart rate (HR) and mean arterial pressure (MAP) are critical indicators for evaluating hemodynamics. In advanced stages of ARDS, patients may exhibit hemodynamic alterations such as unresponsiveness to vasoconstrictors, reduced circulatory vascular resistance, and hypotension. The results of this study demonstrated that patients in the observation group exhibited significantly lower HR and higher MAP compared to the control group post-treatment, indicating that continuous blood purification therapy improves hemodynamics in patients with septic shock complicated by ARDS (32). However, no statistically significant differences were observed in cardiac index (CI). Potential explanations include: first, CI is influenced by both preload and myocardial contractility. While CVVH effectively reduces preload through fluid removal, it does not directly enhance myocardial contractility; moreover, severe ARDS patients often experience impaired contractility due to inflammation-induced myocardial suppression. Second, the included studies may have employed different CI measurement methods (e.g., PiCCO versus echocardiography) and assessment time points (early vs. late stages of CVVH), which could mask true effects. Additionally, patients with normal baseline CI may not show further improvement, resulting in neutral combined outcomes. Clinically, the dissociated phenomenon of improved MAP and HR without significant changes in CI holds particular significance, suggesting that the hemodynamic benefits of CVVH in ARDS patients primarily stem from stabilization of vasomotor function and reduction of afterload rather than increased cardiac output. Therefore, CVVH may be particularly suitable for ARDS patients with concurrent distributive shock or fluid overload; however, its value in patients with confirmed pump failure remains to be determined. Future high-quality randomized controlled trials (RCTs) employing standardized hemodynamic monitoring protocols are required to further elucidate the impact of CVVH on different subtypes of ARDS-related cardiotoxicity (CI). However, only four studies were included and Egger’s test suggested significant small-study effects (*p* = 0.011), so this finding should be interpreted with caution.

This study has certain limitations: ① The geographical specificity of the included studies limits generalizability, as all 28 RCTs were conducted in China with no international multicenter data, potentially leading to regional bias in the results, and variations in the etiology, treatment standards, and CVVH protocols across different regions; ② The follow-up periods in the included literature were insufficient, which may have resulted in underreporting of certain outcome indicators such as mortality rate and recurrence rate, inadequate statistical power, and reporting bias; ③ The evaluation metrics were limited, lacking indicators such as pulmonary dynamic compliance; ④ Due to the lack of information on sepsis status in the majority of included studies (24 out of 28), subgroup analyses based on the presence or absence of sepsis could not be performed, which may have influenced the interpretation of heterogeneity and treatment effects.

In conclusion, CVVH as an adjunctive therapy for ARDS improves oxygenation, reduces mortality, shortens ICU and ventilator days, lowers APACHE II score and heart rate, decreases VAP incidence, and exerts anti-inflammatory and fluid-removing effects. Although substantial heterogeneity and potential publication bias exist (e.g., for oxygenation index and mechanical ventilation duration), and all included studies are from China, CVVH demonstrates promising clinical value. Large-scale, multicenter RCTs are needed to confirm these findings and optimize treatment protocols.

## Glossary

APACHE II: Acute Physiology and Chronic Health Evaluation II
ARDS: Acute Respiratory Distress Syndrome
CI: Cardiac Index
CRP: C-reactive protein
CRRT: Continuous Renal Replacement Therapy
CVVH: Continuous Veno-Venous Hemofiltration
EVLWI: Extravascular Lung Water Index
HR: Heart Rate
ICU: Intensive Care Unit
IL-6: Interleukin-6
MAP: Mean Arterial Pressure
MD: Mean Difference
MODS: Multiple Organ Dysfunction Syndrome
OR: Odds Ratio
PaCO₂: Arterial Carbon Dioxide Partial Pressure
PaO₂: Arterial Oxygen Partial Pressure
PCT: Procalcitonin
PEEP: Positive End-Expiratory Pressure
PiCCO: Pulse Indicator Continuous Cardiac Output
RCT: Randomized Controlled Trial
RRT: Renal Replacement Therapy
SIRS: Systemic Inflammatory Response Syndrome
TNF-*α*: Tumor Necrosis Factor-alpha
VAP: Ventilator-Associated Pneumonia
WBC: White Blood Cell Count
